# Late remission and “late onset” of hypoparathyroidism after total and completion thyroidectomy: a retrospective cohort study of 1060 patients from a tertiary referral center

**DOI:** 10.1007/s12020-025-04330-8

**Published:** 2025-06-30

**Authors:** Sanne Høxbroe Michaelsen, Oke Gerke, Christian Godballe, Steen Joop Bonnema, Viveque Egsgaard Nielsen

**Affiliations:** 1 Research Unit for ORL – Head & Neck Surgery and Audiology, Odense University Hospital, Odense, Denmark; 2 Department of Clinical Research, University of Southern Denmark, Odense, Denmark; 3 OPEN, Open Patient data Explorative Network, Odense University Hospital, Odense, Denmark; 4 Department of Nuclear Medicine, Odense University Hospital, Odense, Denmark; 5 Department of Endocrinology, Odense University Hospital, Odense, Denmark

**Keywords:** Hypoparathyroidism, Parathyroid glands, Prevalence, Thyroidectomy/adverse effects, Postoperative complications/diagnosis, Retrospective studies

## Abstract

**Purpose:**

This single-center retrospective study investigates the incidence of hypoparathyroidism after bilateral thyroidectomy at a tertiary referral center over one decade, including risk factors, time to recovery, and reasons for belated treatment initiation in seemingly “late onset” hypoparathyroidism.

**Methods:**

Patients who underwent total/completion thyroidectomy from January 2011 to December 2020 were included. Potential risk factors for hypoparathyroidism were retrieved from the medical records. Hypoparathyroidism was defined as treatment with an activated vitamin D analogue (AVDA). The incidence was registered 0, 6, 12, and >12 months postoperatively. Hospital records were cross-linked with the national prescription database to ensure complete follow-up.

**Results:**

The incidence of hypoparathyroidism increased from 10.1% upon hospital discharge to 11.4% after 6 months, 12.2% after 12 months, and 12.6% >12 months (median follow-up 7.7 years). The cumulative recovery rate was 19.9% by 6 months, 23.6% by 12 months, and 29.8% >12 months. As many as 23.3% of patients with persistent hypoparathyroidism did not start AVDA treatment within 6 months of surgery, and 12.0% started treatment beyond one year after surgery. In contrast, 9.3% of patients receiving AVDA by 12 months went into remission later on. Two variables emerged as independent risk factors for persistent hypoparathyroidism: re-operation for bleeding (OR 4.21; 95%CI 1.31–13.38) and surgery due to a diagnosis/suspicion of thyroid cancer (OR 2.09; 95%CI 1.04–4.19).

**Conclusion:**

The incidence of postoperative hypoparathyroidism is high and calls for action. Importantly, reassessment of post-surgical parathyroid function should be done regularly in order to neither overlook “late onset” hypoparathyroidism, nor late remission.

## Introduction

Postoperative hypoparathyroidism is the most common complication after bilateral thyroidectomy [1, 2]. The condition ensues when intraoperative injury to the parathyroid glands, or their vasculature, results in a post-surgical level of circulating parathyroid hormone (PTH) which is insufficient to maintain normocalcemia [3, 4]. Postoperative hypoparathyroidism can be either transient or persistent and is often referred to as “permanent” after 6–12 months [5–7]. Nonetheless, a minority of patients may regain normal parathyroid function after more than 12 months [8–11]. The reported incidence of postoperative hypoparathyroidism varies greatly according to the employed definition and clinical setting [12]. An often-cited meta-analysis from 2014 reported a median incidence of permanent post-surgical hypoparathyroidism of just 1% [13]. However, national and multicenter studies report considerably higher rates than single-center studies [14]. To overcome issues such as selection bias and loss to follow-up, larger studies have based their estimations on national prescription or private health insurance databases and defined hypoparathyroidism as “permanent” when ongoing treatment is required 12 months after surgery. This approach has resulted in incidence estimations as high as 12.5–20.3% [15–17]. The heterogeneous definitions of the disorder, and the resulting significant variations in the reported rates of hypoparathyroidism, may mislead both clinicians and patients regarding the risks associated with thyroid surgery [12].

Irrespective of the definition, long-term hypoparathyroidism has serious implications for the individual patient [18, 19]. Consequently, surgeons should be keenly aware of both patient-, disease-, and surgery-related risk factors for the development of postoperative hypoparathyroidism [5, 20]. While risk factors for transient hypoparathyroidism have been relatively well-described, studies on risk factors for permanent hypoparathyroidism are characterized by a low number of events (i.e. the number of patients with permanent hypoparathyroidism) relative to the number of predictor variables being tested [9, 13, 21]. In a recent systematic review and meta-analysis of univariate associations between potential risk factors and permanent hypoparathyroidism, only 9 of 85 studies reported more than 100 events, and 3 of these 9 studies were even based on the same patient cohort [15, 21–23]. Accordingly, the authors found considerably fewer statistically significant risk factors for permanent hypoparathyroidism in the literature compared with transient hypoparathyroidism [21].

The aim of this study was to investigate the incidence and timing of postoperative hypoparathyroidism 0, 6, 12 and >12 months after bilateral thyroidectomy in a cohort of a sufficient size to meaningfully assess potential risk factors for persistent hypoparathyroidism. The study covers a 10-year period at a tertiary referral center and furthermore examines the time to recovery of transient hypoparathyroidism as well as underlying causes of delayed treatment in cases initially classified as “late onset” hypoparathyroidism.

## Materials and methods

### Study design

This was a single-institution, retrospective review of all patients who underwent total or completion thyroidectomy from January 1, 2011 to December 31, 2020 at the Department of Otorhinolaryngology, Head & Neck Surgery at Odense University Hospital, Denmark. The study was approved by the Region of Southern Denmark with journal no. 20/50700. Reporting guidelines from Strengthening the Reporting of Observational Studies in Epidemiology (STROBE) were employed [24].

### Identification of potentially eligible patients

Patients who underwent total thyroidectomy were identified by the procedure code. However, completion thyroidectomies did not have a unique procedure code. Consequently, patients who potentially underwent completion procedures were identified for further review if they had: a) two procedure codes for a hemithyroidectomy within the study period, or b) a single procedure code plus at least one prior registration of thyroid tissue in the national Danish Pathology Data Bank. Relevant pathology reports were subsequently reviewed for contralateral thyroid lobectomies.

### Exclusion criteria

The medical records of all identified patients were screened for the following exclusion criteria: no total or completion thyroidectomy within the study period; intentional parathyroidectomy performed as a supplementary procedure (including surgery for primary/tertiary hyperparathyroidism); chronic kidney disease (estimated glomerular filtration rate <30 mL/min); preoperative treatment with an activated vitamin D analogue (AVDA); postoperative treatment with an AVDA for reasons other than iatrogenic parathyroid injury.

### Demographic and clinical data

From the patient records, we extracted data on the variables listed in Table 1 as well as available blood tests. Surgical indication was divided into three categories: 1) compressive symptoms, 2) hyperthyroidism (Graves’ disease, toxic nodular goiter, or hyperfunctioning thyroid nodules), and 3) a diagnosis or suspicion of thyroid malignancy. The surgeries of patients from the third category adhered to a standardized national protocol known as a “cancer patient pathway” [25].

**Table 1:** Clinical and demographic characteristics

		Total	Never AVDA	Ever AVDA	p-value
Variable (unit)		N = 1060 (100%)	N = 869 (82%)	N = 191^a^ (18%)	
**Sex**	male	292 (28%)	240 (28%)	52 (27%)	0.91
	female	768 (72%)	629 (72%)	139 (73%)	
**Age** (years)		54 (42–66)	54 (42–67)	52 (38–62)	**0.007**
**Body mass index**^b^ (kg/m^2^)		26.9 (23.7–30.7)	27.1 (23.7–30.8)	26.3 (22.7–30.4)	**0.049**
**Procedure**					**0.006**
total thyroidectomy		686 (65%)	546 (63%)	140 (73%)	
completion thyroidectomy		374 (35%)	323 (37%)	51 (27%)	
**Surgical indication**					
compressive symptoms		339 (32%)	299 (34%)	40 (21%)	**<0.001**
hyperthyroidism		160 (15%)	118 (14%)	42 (22%)	
diagnosed/suspected cancer		561 (53%)	452 (52%)	109 (57%)	
**Substernal goiter**	no	896 (85%)	731 (84%)	165 (86%)	0.43
	yes	164 (15%)	138 (16%)	26 (14%)	
**Former neck surgery**	no	994 (94%)	818 (94%)	176 (92%)	0.30
	yes	66 (6%)	51 (6%)	15 (8%)	
**Lymph node excision/neck dissection**	no	884 (83%)	738 (85%)	146 (76%)	**0.004**
	yes	176 (17%)	131 (15%)	45 (24%)	
**Operating surgeon**^**c**^		n = 7	n = 7	n = 7	0.75
**Malignant histopathology**	no	529 (50%)	441 (51%)	88 (46%)	0.24
	yes	531 (50%)	428 (49%)	103 (54%)	
**Weight of both thyroid lobes**^d^ (g)		57 (28–126)	58 (28–128)	53 (26–115)	0.30
**Duration of surgery**^e^ (minutes)		76 (59–103)	74 (57–100)	88 (66–118)	**<0.001**
**Perioperative blood loss**^f^ (ml)		40 (20–96)	38 (20–85)	50 (22–121)	**0.003**
**Reoperation for bleeding**	no	1044 (98.5%)	861 (99%)	183 (96%)	**<0.001**
	yes	16 (1.5%)	8 (1%)	8 (4%)	
**≥1 Autotransplanted parathyroid gland**	no	958 (90%)	787 (91%)	171 (90%)	0.66
	yes	102 (10%)	82 (9%)	20 (10%)	
**≥ 1 Parathyroid glands on histopathology**	no	866 (82%)	719 (83%)	147 (77%)	0.062
	yes	194 (18%)	150 (17%)	44 (23%)	
**Transient recurrent laryngeal nerve palsy <12 months**^**g**^	no	1009 (95.2%)	833 (95.9%)	176 (92.1%)	**0.030**
	yes	51 (4.8%)	36 (4.1%)	15 (7.9%)	
**Recurrent laryngeal nerve palsy** **>** **12 months**^**g**^	no	1035 (97.6%)	851 (97.9%)	184 (96.3%)	0.19
	yes	25 (2.4%)	18 (2.1%)	7 (3.7%)	
**Nights in hospital**		2 (2–3)	2 (2–3)	4 (3–5)	**<0.001**

Data are presented as median (interquartile range) for continuous measures, and number (%) for categorical measures; Bold writing indicates statistical significance; AVDA: treatment with an active vitamin D agonist

^a^Not including 4 patients who both started and stopped treatment in-between the pre-defined follow-up dates

^b^34 missing values

^c^This category consists of six individual experienced surgeons and a pooled group of low volume surgeons (e.g. supervised residents)

^d^158 missing values

^e^7 missing values

^f^126 missing values

^g^Indirect or fiberoptic laryngoscopy

### Primary outcome

In Denmark, treatment of hypoparathyroidism is managed by endocrinologists and usually consists of an AVDA combined with calcium supplementation [26, 27]. Systematic attempts at medication tapering are standard procedure [26]. Because complete biochemical data were not available for the entire cohort, a filled prescription for an AVDA (Anatomical Therapeutic Chemical classification system codes A11CC02, A11CC03 or A11CC04) served as a surrogate marker for postsurgical hypoparathyroidism. This approach complies with the practice of the Danish national Thyroid Database and a European expert consensus from 2022, which stresses the need for criteria that enable incidence comparisons between different countries [28, 29]. Accordingly, patients not treated with an AVDA will be referred to as euparathyroid in this text. AVDA treatment status was registered at four time points: upon discharge, 6 months after surgery, 12 months after surgery, and the most recent data. To ensure complete follow-up regarding the primary outcome, AVDA treatment status was cross-linked with The Danish National Prescription Registry, which contains complete and valid individual-level data on prescriptions redeemed at community pharmacies in Denmark since 1995 [30]. The cut-off date from the registry was December 31, 2023.

### Secondary outcomes

Secondary outcomes were a) potential risk factors for postoperative hypoparathyroidism, b) the time to recovery and characteristics of patients with transient hypoparathyroidism, and c) reasons for “late onset” treatment initiation with an AVDA ( >6 months).

### Surgery

Autotransplantation of seemingly devascularized parathyroid glands was performed at the discretion of the surgeon. Thyroidectomies performed due to a suspicion of malignancy were carried out in accordance with the existing Danish national guideline at the time of surgery (version 2010 or 2015), which was strongly influenced by guidelines from the American Thyroid Association [31, 32]: According to the guideline, an initial hemithyroidectomy with lymph node exploration of level VI was performed if a fine needle aspiration biopsy (FNAB) from a scintigraphically cold/FDG-PET positive thyroid nodule was indeterminate or suspicious for malignancy (FDG-PET: fluorodeoxyglucose positron emission tomography). A completion/total thyroidectomy with exploration/dissection of level VI was supplied if histopathology revealed: a thyroid carcinoma metastasis; aggressive histology; penetration of the thyroid capsule; a carcinoma >2 cm; or a multifocal microcarcinoma with either >3 foci or a total diameter >1 cm (2015 guidelines). If metastases were suspected, either perioperatively or from pre-operative imaging/cytology, the involved and adjacent neck levels were dissected, except for level 1 (2015 guidelines). The 2010 guidelines were slightly more conservative (e.g. any multifocal tumor, or a papillary tumor >1 cm, would result in a completion/total thyroidectomy).

### Postoperative management

Plasma ionized calcium (Ca^2+^) was measured on the night of the surgery and twice daily on subsequent days until increasing or stable values were reached. Ca^2+^ was measured with standard laboratory methods adjusted to a pH of 7.4 (reference level 1.18–1.32 mmol/l). PTH measurements were not standard practice at the time, but the hospital employed a second generation PTH assay until 2019 (Siemens Immulite 2000 Intact PTH) and a third generation PTH assay from 2019 and onwards (Roche Cobas e 801 Elecsys PTH 1–84). If calcium levels continually declined despite calcium supplementation, an AVDA was added. Patients with a malignant diagnosis received follow-up at the regional department of oncology. Patients with benign histology and patients treated with an AVDA at discharge (also) received follow-up at their local department of endocrinology.

### Death during follow-up

The few patients who died <12 months after surgery while being treated with an AVDA were evaluated based on their latest biochemistry and the duration of follow-up. If a biochemically hypercalcemic patient died <3 months after surgery, the long-term prognosis was considered unresolved and the patient was excluded from subsequent incidence analyses. In all other cases, the patient was considered to have had persistent hypoparathyroidism.

### Statistics

No continuous variables were normally distributed (Shapiro Wilk’s test). Pearson’s χ^2^ test (categorical variables) or the Wilcoxon rank sum test (continuous variables) were used for comparing independent samples. Multivariable analysis was conducted using binary logistic regression [33]. Candidate variables were selected based on their distribution and clinical knowledge, with a lower limit of ten events per candidate variable [7, 13, 34]. The distribution of missing values did not necessitate the exclusion of any variable from the multivariable analysis. Candidate variables are listed in Table 2. The subsequent variable reduction was performed by backward elimination starting from the highest p-value, with an increase in Akaike’s Information Criterion as the stop criterion [34]. Histopathological malignancy was not included as a candidate variable due to being closely related to surgical indication. The variable “operating surgeon” comprised six individual experienced thyroid surgeons and a seventh pooled reference category called “low volume thyroid surgeon” consisting of surgeons with a yearly average <10 total/completion thyroidectomies – e.g. supervised residents or new additions to the surgical team [35]. Damage to the recurrent laryngeal nerve was included based on the hypothesis that a loss of signal on the perioperative neural monitor would divert attention away from parathyroid preservation.

**Table 2:** Univariable analysis showing the distribution of potential risk factors between normo- and hypoparathyroid patients

		Hypoparathyroid at discharge			Hypoparathyroid at 6 months			Hypoparathyroid at 12 months			Hypoparathyroid > 12 months		
		no	yes	p-value	no	yes	p-value	no	yes	p-value	no	yes	p-value
**Variable** (unit)		N = 953	N = 107		N = 938	N = 121		N = 930	N = 129		N = 926	N = 133	
**Sex**	male	266 (28%)	26 (24%)	0.43	260 (28%)	32 (26%)	0.77	257 (28%)	35 (27%)	0.90	255 (28%)	37 (28%)	0.95
	female	687 (72%)	81 (76%)		678 (72%)	89 (74%)		63 (72%)	94 (73%)		671 (72%)	96 (72%)	
**Age** (years)		55 (43–67)	50 (34–58)	**<0.001**	54 (42–67)	51 (39–62)	**0.028**	54 (42–67)	51 (42–62)	0.12	54 (42–67)	51 (42–62)	0.099
**Body mass index**^a^ (kg/m2)		27.1 (23.7–30.8)	25.7 (21.9–29.7)	**0.019**	27.0 (23.7–30.7)	26.7 (23.1–30.8)	0.58	27.1 (23.7–30.8)	26.3 (22.9–30.1)	0.25	27.1 (23.7–30.8)	26.4 (23.2–30.4)	0.40
**Procedure**				**<0.001**			0.078			0.48			0.70
total thyroidectomy		592 (62%)	94 (88%)		598 (64%)	87 (72%)		598 (64%)	86 (67%)		597 (64%)	88 (66%)	
completion thyroidectomy		361 (38%)	13 (12%)		340 (36%)	34 (28%)		332 (36%)	42 (33%)		329 (36%)	45 (34%)	
**Surgical indication**				**<0.001**			**0.005**			**0.008**			**0.004**
compressive symptoms		319 (33%)	20 (19%)		314 (33%)	25 (21%)		313 (34%)	26 (20%)		313 (34%)	26 (20%)	
hyperthyroidism		126 (13%)	34 (32%)		133 (14%)	27 (22%)		136 (15%)	24 (19%)		138 (15%)	22 (17%)	
diagnosed/suspected cancer		508 (53%)	53 (50%)		491 (52%)	69 (57%)		481 (52%)	79 (61%)		475 (51%)	85 (64%)	
**Substernal goiter**	no	802 (84%)	94 (88%)	0.32	789 (84%)	107 (88%)	0.22	784 (84%)	112 (87%)	0.46	780 (84%)	116 (87%)	0.37
	yes	151 (16%)	13 (12%)		149 (16%)	14 (12%)		146 (16%)	17 (13%)		146 (16%)	17 (13%)	
**Former neck surgery**	no	892 (94%)	102 (95%)	0.48	881 (94%)	112 (93%)	0.56	875 (94%)	118 (91%)	0.25	871 (94%)	122 (92%)	0.30
	yes	61 (6%)	5 (5%)		57 (6%)	9 (7%)		55 (6%)	11 (9%)		55 (6%)	11 (8%)	
**Lymph node excision/neck dissection**	no	805 (84%)	79 (74%)	**0.005**	789 (84%)	94 (78%)	0.074	786 (85%)	97 (75%)	**0.008**	784 (85%)	99 (74%)	**0.003**
	yes	148 (16%)	28 (26%)		149 (16%)	27 (22%)		144 (15%)	32 (25%)		142 (15%)	34 (26%)	
**Operating surgeon**^b^		n = 7		0.95	n = 7		0.29	n = 7		0.14	n = 7		0.29
**Malignant histopathology**	no	471 (49%)	58 (54%)	0.35	474 (10%)	55 (45%)	0.29	475 (51%)	54 (42%)	**0.0498**	475 (51%)	54 (41%)	**0.021**
	yes	482 (51%)	49 (46%)		464 (49%)	66 (55%)		455 (49%)	75 (58%)		451 (49%)	79 (59%)	
**Thyroid weight**^c^ (g)		58 (27–128)	52 (30–115)	0.55	58 (27–127)	52 (29–125)	0.40	58 (28–128)	48 (26–105)	0.19	58 (28–128)	46 (23–104)	0.079
**Duration of surgery**^d^ (minutes)		73 (57–100)	97 (78–126)	**<0.001**	75 (58–101)	88 (66–119)	<**0.001**	75 (58–101)	88 (64–122)	**0.002**	75 (58–101)	89 (64–121)	**0.001**
**Blood loss**^e^ (ml)		39 (20–85)	56 (32–145)	**<0.001**	40 (20–90)	50 (22–120)	**0.030**	40 (20–86)	50 (21–145)	**0.013**	40 (20–90)	48 (20–120)	0.20
**Reoperation for bleeding (ml)**	no	940 (99%)	104 (97%)	0.25	928 (99%)	115 (95%)	**<0.001**	921 (99%)	122 (95%)	**<0.001**	918 (99%)	125 (94%)	**<0.001**
	yes	13 (1%)	3 (3%)		10 (1%)	6 (5%)		9 (1%)	7 (5%)		8 (1%)	8 (6%)	
**Recurrent laryngeal nerve palsy**^**f**^	no	912 (96%)	97 (91%)	**0.021**	896 (96%)	112 (93%)	0.15	889 (96%)	119 (92%)	0.096	886 (96%)	122 (92%)	**0.047**
	yes	41 (4%)	10 (9%)		42 (4%)	9 (7%)		41 (4%)	10 (8%)		40 (4%)	11 (8%)	
**≥1 Autotransplanted parathyroid glands**	no	864 (91%)	94 (88%)	0.35	847 (90%)	110 (91%)	0.83	839 (90%)	118 (91%)	0.65	831 (90%)	126 (95%)	0.068
	yes	89 (9%)	13 (12%)		91 (10%)	11 (9%)		91 (10%)	11 (9%)		95 (10%)	7 (5%)	
**≥1 Parathyroid gland on histopathology**	no	777 (82%)	89 (83%)	0.68	769 (82%)	97 (80%)	0.63	767 (82%)	99 (77%)	0.11	768 (83%)	98 (74%)	**0.010**
	yes	176 (18%)	18 (17%)		169 (18%)	24 (20%)		163 (18%)	30 (23%)		158 (17%)	35 (26%)	

Data are median (interquartile range) for continuous measures and number (%) for categorical measures; Bold writing indicates statistical significance

^a^34 missing

^b^Six individual experienced surgeons and a pooled group of low volume surgeons (e.g. supervised residents)

^c^Weight of both thyroid lobes 158 missing

^d^7 missing

^e^126 missing

^f^Transient or permanent

Study data were managed with REDCap® electronic data capture tools hosted at Odense University Hospital [36, 37]. Statistical calculations were conducted in StataNow/BE: release 18.5 and Stata/MP 18.0 (StataCorp LLC).

## Results

### Patient characteristics

**Fig. 1:**
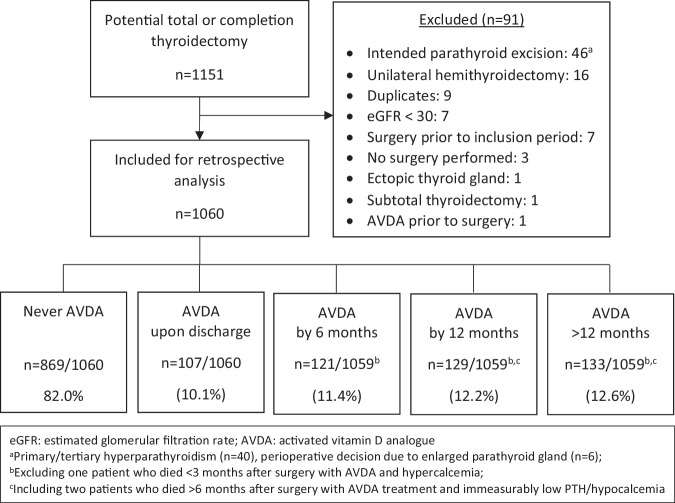
Flow diagram including the incidence of hypoparathyroidism over time

A total of 1,060 patients were included in the retrospective analysis (Fig. 1). Total thyroidectomy was the most common procedure (65%) and the prevailing surgical indication was a diagnosis/suspicion of cancer (53%) (Table 1). The majority of patients with hyperthyroidism had Graves’ disease (83%, n = 133/160). Follow-up was complete in all patients with a median of 7.7 years (range: 3–13 years). Three patients died <12 months while treated with an AVDA, one with hypercalcemia and two with immeasurably low PTH/hypocalcemia. Furthermore, one hyperparathyroid patient started AVDA treatment due to the progression of diabetic nephropathy and was not considered to have surgically induced hypoparathyroidism. Patient characteristics are summarized in Table 1.

### Primary outcome

Figure 1 shows the patient selection flow chart along with the percentage of patients with hypoparathyroidism 0, 6, 12, and >12 months after surgery. Cumulatively, 18.0% of all patients (n = 191/1060) received treatment with an AVDA at any of the pre-defined time points (Table 1). The largest incidence of postsurgical hypoparathyroidism occurred >12 months after surgery (12.6%, n = 133/1059).

### Secondary outcomes

#### Risk factors

The results of the univariable analysis are displayed in Table 2. Surgical indication and duration of surgery differed significantly between hypo- and euparathyroid patients at all evaluated time points after surgery.

On multivariable analysis, four variables emerged as significant risk factors for developing postoperative hypoparathyroidism (Table 3): Re-operation for bleeding and surgery due to a diagnosis/suspicion of thyroid cancer were risk factors for hypoparathyroidism with a duration >12 months. Surgery due to hyperthyroidism was a risk factor for having hypoparathyroidism from hospital discharge and until one year after surgery, but not for persistent hypoparathyroidism >12 months. Total thyroidectomy was a risk factor for transient hypoparathyroidism ( ≤6 months after surgery).

**Table 3:** Multivariable analysis of potential risk factors for postoperative hypoparathyroidism

	At discharge		6 months		12 months		>12 months	
	OR	95% CI	OR	95% CI	OR	95% CI	OR	95% CI
**Body mass index** (kg/m^2^)	0.96	0.92–1.01	0.998	0.96–1.04	0.99	0.95–1.03	0.99	0.95–1.03
**Procedure:** total thyroidectomy^a^	4.72	**1.97**–**11.30**	2.19	**1.12**–**4.26**	1.43	0.75–2.74	1.32	0.73–2.39
**Surgical indication**^b^								
hyperthyroidism	3.49	**1.74**–**6.98**	2.29	**1.15**–**4.57**	2.16	**1.06**–**4.39**	1.75	0.85–3.61
diagnosed/suspected cancer	2.31	**1.01**–**5.28**	1.78	0.85–3.73	1.89	0.87–4.12	2.19	**1.07**–**4.47**
**Former central neck surgery**	0.82	0.30–2.26	0.88	0.34–2.26	1.17	0.50–2.76		
**Lymph node excision/neck dissection**	1.59	0.76–3.31			1.26	0.64–2.48		
**Operating surgeon**^c^								
Surgeon 1	0.53	0.15–1.89	0.50	0.15–1.73	0.48	0.14–1.64	0.45	0.13–1.54
Surgeon 2	0.50	0.17–1.49	0.51	0.18–1.46	0.54	0.19–1.55	0.57	0.20–1.63
Surgeon 3	0.46	0.14–1.55	0.40	0.12–1.35	0.32	0.09–1.12	0.38	0.11–1.29
Surgeon 4	0.35	0.09–1.26	0.43	0.13–1.46	0.53	0.16–1.73	0.58	0.18–1.87
Surgeon 5	0.53	0.17–1.71	0.75	0.25–2.27	0.75	0.25–2.23	0.55	0.18–1.64
Surgeon 6	0.38	0.10–1.44	0.38	0.10–1.42	0.38	0.10–1.38	0.29	0.08–1.09
**Thyroid weight** (g)	1.0004	0.997–1.003	0.999	0.996–1.002	0.999	0.996–1.002	0.999	0.996–1.002
**Duration of surgery** (minutes)	1.001	0.999–1.004	1.001	0.998–1.004	1.001	0.998–1.004	1.001	0.998–1.004
**Blood loss** (ml)	1.001	0.9995–1.002	1.0004	0.999–1.001	1.001	0.9996–1.002	1.001	0.9997–1.002
**Reoperation for bleeding**			3.14	0.89–11.03	3.91	**1.18**–**12.95**	4.33	**1.33**–**14.08**
**RLN nerve palsy**^d^	1.34	0.52–3.42	1.25	0.47–3.30	1.40	0.56–3.49	1.87	0.79–4.44

Blank space: variable removed from model using backward elimination guided by p-values and Akaike’s information criterion; Bold writing indicates statistical significance; *OR* odds ratio, *CI* confidence interval, *RLN* recurrent laryngeal nerve

^a^Completion thyroidectomy is the reference category

^b^Compressive symptoms is the reference category

^c^Low volume surgeon is the reference category

^f^Transient or permanent

#### Time to recovery

**Fig. 2:**
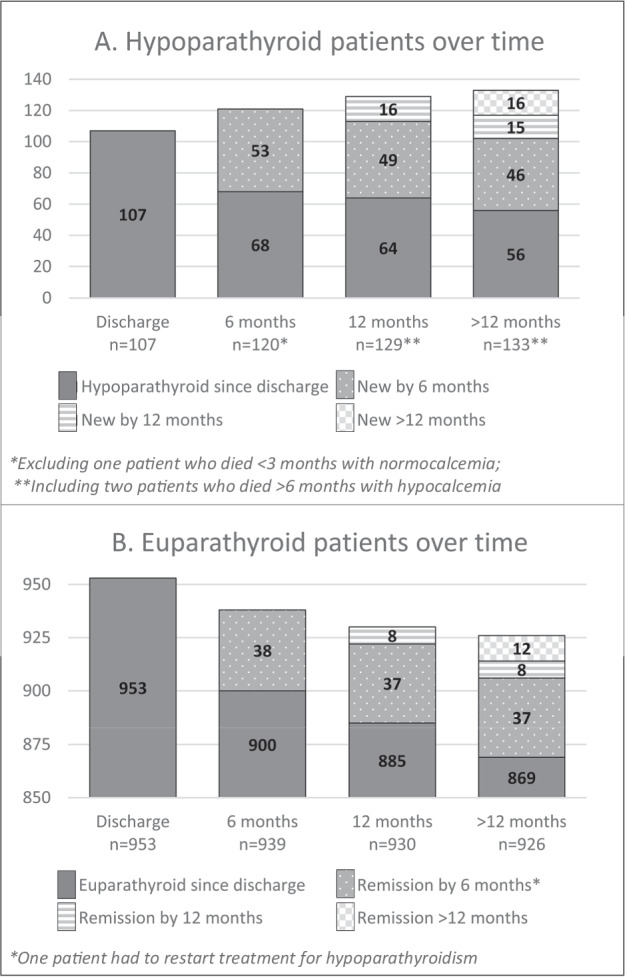
Evolution of parathyroid function over time

Figure 2 shows the evolution of parathyroid function over time. Of the 191 patients treated with an AVDA at any time after surgery, the median treatment duration was 4.7 years. The cumulative recovery rate was 19.9% (n = 38/191) by 6 months, 23.6% (n = 45/191) by 12 months, and 29.8% (n = 57/191) beyond 12 months. Of the 12 patients who took more than one year to recover, the minimum recovery time was 12.8 months, the median 1.8 years, and the maximum 7.8 years. A revision of the medical files confirmed the 7.8-year duration, including attempts at tapering and pausing the medication. All 12 patients had plasma PTH levels within the normal range, while ionized calcium ranged from 1.11–1.26 (median 1.17) mmol/L.

#### Characteristics of patients who recovered

There was no association between surgical indication and time to recovery (p = 0.789 for recovery within six months and p = 0.338 for recovery within one year of surgery). Autotransplantation was nominally more frequent among patients who took more than 12 months to recover (41.7%, n = 5/12) compared with those who took less than 12 months to recover (17.8%, n = 8/37), but the difference was insignificant (p = 0.080). Among the patients who were ever treated with an AVDA after surgery, the rate of parathyroid autotransplantation was significantly higher in those who recovered (22.8%, n = 13/57) compared with those who never recovered (5.26%, n = 7/133, p < 0.001). None of the patients with late recovery ( >12 months) developed clinical complications of hypoparathyroidism (e.g. chronic kidney disease) before their remission.

#### Reasons for belated treatment initiation

A total of 32 patients started AVDA treatment more than 6 months after surgery (Fig. 2A). Fifteen (46.9%) cases were due to a late referral from the oncological department to the department of endocrinology, 8 (25%) due to a longer period of watchful waiting on calcium supplements only, 2 (6.3%) due to the patient’s reluctance to start treatment, and 7 (21.9%) for unclear reasons.

## Discussion

In this study, we provide one decade’s worth of data on iatrogenic hypoparathyroidism after bilateral thyroidectomy at a tertiary referral center with complete follow-up regarding the primary outcome. We made four principal findings: 1) The incidence of AVDA-treated hypoparathyroidism continually increased, reaching 12.6% more than one year after surgery; 2) Re-operation for bleeding and surgery due to a diagnosis/suspicion of cancer were independent risk factors for persistent hypoparathyroidism; 3) As many as 23.3% of patients with persistent hypoparathyroidism did not start AVDA treatment within 6 months of surgery, and 12.0% did not start treatment within one year of surgery; 4) As many as 9.3% of patients receiving AVDA 12 months after surgery went into remission later on.

The incidence of iatrogenic hypoparathyroidism in our cohort is in concordance with a growing number of studies that show a high and non-negligible rate of long-term postsurgical hypoparathyroidism [15–17, 38–41]. Despite of this, we had to consider whether the high incidence in our cohort was due to lacking surgical standards. However, any such lack is not reflected in the complication rates for postoperative bleeding and recurrent laryngeal nerve paralysis, which are in line with a national registry study from the United Kingdom (1.6% postoperative bleeding) and a meta-analysis of 25,000 thyroidectomy patients (2.3% recurrent nerve palsy) [42, 43]. Furthermore, our rate of hypoparathyroidism is lower than the 16.6% reported in a recent nationwide cohort study from Denmark, which employed an identical definition of hypoparathyroidism, but did not examine risk factors [16]. A reason for this discrepancy could be that the nationwide study included low volume centers with rates of long-term hypoparathyroidism as high as 31.5% [44].

In the multivariable analysis, re-operation for bleeding and surgery due to a diagnosis/suspicion of cancer emerged as the only independent risk factors for persistent hypoparathyroidism. This is in keeping with a Swedish multicenter study from 2012, which identified postoperative bleeding as an independent long-term risk factor (odds ratio 10.59) [45]. This finding underlines that surgeons should strive to preserve the parathyroid feeding vessels even during emergency hemostasis procedures [46]. When it comes to a diagnosis/suspicion of cancer as an independent risk factor for hypoparathyroidism, previous studies have reached inconsistent conclusions [10, 21]. However, most analyses are based on the postoperative histopathology report rather than on the information provided to the surgeon at the onset of surgery. We would argue, though, that an unforeseen histopathological diagnosis of cancer in assumed benign disease is unlikely to have affected the surgical procedure. Conversely, surgery due to a suspicion of cancer will justifiably make the surgeon hesitant to leave a potential parathyroid gland in situ if there is any doubt that the identified structure could be a lymph node metastasis. Future studies should consider making this distinction as well. Finally, our study identified hyperthyroidism (83% Graves’ disease) as an independent risk factor for postoperative hypoparathyroidism lasting up to, but not beyond, 12 months. Graves’ disease is a known risk factor for transient postoperative hypoparathyroidism, but the risk for long-term disease remains unclear [13]. For example, a large study of 5845 patients reported an odds ratio of 2.4 for hypoparathyroidism >6 months in patients with Graves’ disease, whereas a recent 1965 patient series found no significant increase in hypoparathyroidism 12–80 months after surgery in patients with “autoimmune thyroiditis” [9, 47].

A curious finding in our study is the large percentage of patients with persistent hypoparathyroidism who did not start treatment within 12 months after surgery. This group is rarely addressed in the literature. In fact, retrospective studies on long-term postsurgical hypoparathyroidism have been known to only follow-up on patients who were hypoparathyroid 1–3 months after surgery [41, 48]. By comparison, if we had only reviewed the medical records of patients known to be hypoparathyroid at discharge, the reported incidence of persistent hypoparathyroidism would have been 5.3% (56/1059) instead of 12.6% (133/1059) (Fig. 2A). Similarly, if we had only followed-up on patients who were hypoparathyroid 6 months after surgery, we would have missed 31 (23.3%) of the 133 patients with hypoparathyroidism >12 months (Fig. 2A). This shows that not only the definition of hypoparathyroidism, but also the method of follow-up in retrospective studies can lead to considerably different results, thereby contributing to the large variation in reported incidences of postoperative hypoparathyroidism [12]. In our cohort, belated treatment initiation most frequently occurred in patients with malignant histopathology who did not display symptoms necessitating AVDA treatment immediately after surgery. These patients were referred directly to the department of oncology and did not receive a timely referral to an endocrinologist despite presenting persistently low plasma ionized calcium levels during their oncological follow-up. This problem could likely be resolved with a greater focus on postsurgical hypoparathyroidism among oncologists, or with routine postoperative PTH measurements before hospital discharge to ensure an early diagnosis of hypoparathyroidism. The second most common reason was intentional “watchful waiting” in patients with parathyroid insufficiency who were assumed to recover based on normal or near-normal PTH levels. Finally, a number of studies have hypothesized that adjuvant cancer treatments, such as radioactive iodine, may have an influence on the risk of developing late-onset hypoparathyroidism. However, the studies have reached dissimilar conclusions [49, 50].

Similarly to other studies, our data imply that hypoparathyroidism should not necessarily be considered permanent one year after surgery [8–11]. There could be more than one explanation for this. First, devascularized parathyroid glands may take longer than 12 months to regain a sufficient hormone production [51]. Second, inconsistent patient adherence to treatment or control regimens may prolong periods of observation before medication tapering. Third, medication tapering may take several months, depending on the clinical practice [52]. By way of example, an international current practice survey from 2022 showed that approximately 40% of the responding clinicians performed follow-up of patients with nonstable chronic postoperative hypoparathyroidism every three months or less frequently [53]. For patients with stable disease, approximately 60% of respondents performed follow-up with six-month intervals or less frequently.

A number of protective measures can be undertaken to minimize harm to the parathyroid glands during thyroid surgery. These include gentle capsular thyroid dissection, autotransplantation of inadvertently resected parathyroid glands, loupe magnification, and keen awareness of the zone of collateral thermal spread from electrocautery devices [7]. The high incidence of persisting postsurgical hypoparathyroidism, however, reveals that further efforts are needed [16]. Emerging fluorescent imaging technologies hold promise to decrease postsurgical hypoparathyroidism by aiding the surgeon in identifying the parathyroid glands, preserving their feeding vessels, and guiding intraoperative decision-making [54]. Although initial results have been encouraging, these optical aides have yet to demonstrate significant decreases in long-term hypoparathyroidism [55, 56].

The following limitations should be considered: This was a single-center study from a university hospital in a Nordic country with tax-supported free access to healthcare, and the generalizability of the results may be limited to similar settings. The rationale for including both total thyroidectomies and completion thyroidectomies in our cohort was that the patients in both groups have had bilateral thyroid lobectomies, which entails risk of iatrogenic injury to all four parathyroid glands. Furthermore, 85% of the completion lobectomies were performed within a few weeks of the initial hemithyroidectomy due to histopathologically verified thyroid cancer in the contralateral lobe. However, the inclusion of completion thyroidectomies in the cohort might underestimate the effect of the variable “neck dissection/lymph node excision” on the risk of developing postoperative hypoparathyroidism. We tried to ameliorate the effect of this limitation by dichotomizing the data regarding neck dissection/lymph node excision to yes/no, reasoning that a patient with nodal disease on histopathology from the first hemithyroidectomy would at least undergo central neck dissection during the completion procedure. Also, the initial lobectomy in completion procedures was not always performed at our department. We estimated the effect hereof by repeating the multivariable analysis after exclusion of the patients for whom we did not know the identity of the surgeon from the first lobectomy (n = 66). Doing so did not change the significance of any potential risk variable at any postoperative time point. In fact, previous studies have reported similar complication rates between one-stage total thyroidectomy and two-stage completion procedures, although the rate of temporary hypoparathyroidism appears to be more frequent for one-stage procedures, in accordance with our results [57, 58]. Even though our study has a comparatively large number of events, it is possible that risk factors of lesser magnitude could have been missed due to low statistical power. Because of the retrospective nature of the study, we were unable to biochemically corroborate that all patients required the treatment they received. However, the 3–13 year follow-up period increases the likelihood that unnecessary treatment would eventually have been discontinued. Furthermore, our study used treatment with an AVDA to diagnose hypoparathyroidism. Many other studies accept the use of calcium supplements, with or without AVDA, as a marker of hypoparathyroidism [1, 15]. However, hypoparathyroidism that does not require treatment with an AVDA is unlikely to be clinically significant. In addition, ordinary over-the-counter calcium and vitamin-D supplements (excluding AVDA) are not registered by the national prescription database and are taken by many people for various reasons. For instance, all Danish citizens above the age of 70, as well as patients at risk of osteoporosis regardless of age, are recommended by the Danish Health Authority to take calcium and vitamin D supplements [59]. Finally, the cross-link with the national prescription database has ensured complete follow-up, which is a major strength of the study.

## Conclusion

In our cohort, reoperation for bleeding and a diagnosis/suspicion of cancer were individual risk factors for persistent ( >12 months) postsurgical hypoparathyroidism. We found a high and non-negligible incidence of hypoparathyroidism both 6, 12, and >12 months after surgery. In light of the serious health implications of permanent hypoparathyroidism, these numbers require action. New technologies and refined surgical techniques may help achieve this goal, but further research is necessary [54]. Since hypoparathyroidism may recover beyond 12 months after surgery, reassessment of parathyroid function should be done regularly to enable appropriate tapering of AVDA. Similarly, in order to avoid unnecessary treatment delays for patients with low postoperative calcium, both surgical and oncological staff should make timely referrals to the department of endocrinology [60]. Lastly, each institution should assess their own rate of postsurgical hypoparathyroidism in order to provide patients with realistic risk estimates before surgery.

## Data Availability

No datasets were generated or analysed during the current study.
